# Development-centred neonatal care: validation study of the patient safety checklist

**DOI:** 10.3389/fped.2026.1754878

**Published:** 2026-05-12

**Authors:** Ana Malveira, Marília Teles, Leonel Lusquinhos, Ana Dias, Sandra Oliveira, Manuel Lopes

**Affiliations:** 1 Unidade Local de Saúde ALentejo Central, Neonatal Intensive Care Unit (NICU), Évora, Portugal; 2 Nursing Research, Innovation and Development Centre of Lisbon (CIDNUR), School of Nursing, Universidade de Lisboa, Lisbon, Portugal; 3 Comprehensive Health Research Centre (CHRC), Évora, Portugal; 4 Universidade de Évora, São João de Deus School of Nursing, Évora, Portugal; 5 Centre for Health Studies and Research, Universidade de Coimbra (CEISUC), Coimbra, Portugal; 6 Santarém Polytechnic University, School of Management and Technology, Santarém, Portugal

**Keywords:** checklist, intensive care, neonatal, patient safety, patient-Centred care, quality indicator, healthcare

## Abstract

**Objectives:**

The Neonatal Patient Safety Checklist (NPSC) is a quality tool designed to assess compliance with care interventions by defining and operationalising neonatal quality and safety indicators. While many indicators have previously been validated and implemented in various international safety checklists, the NPSC is unique in including a set of safety indicators related to development-centred care (DCC), ensuring the quality and safety dimensions advocated by person-centred care. The aim of this study was to validate the NPSC indicators by ensuring consensus through representativeness and relevance to the neonatal care context.

**Methodology:**

The Delphi method was selected for this study. A sample of 19 experts participated in two rounds of structured questionnaires, which were administered individually in sequence. Researchers analysed and systematised the responses from each round, providing feedback in the form of a synthesis of the group's previous responses. This process aimed to develop a cumulative collective response and ensure a consensual construct.

**Results:**

Of the initial 56 indicators, only four had a content validity index (CVI) < 80%, indicating that the construct was not adequately represented. However, the qualitative input of the expert panel enabled the remaining indicators to be reformulated, achieving a CVI of at least 85.7%. This demonstrates that the NPSC is a robust, representative tool for neonatal care, with consensus achieved among experts.

**Conclusions:**

Implementing this methodology resulted in the creation of a tool strongly linked to quality and safety. This was achieved by operationalising a set of innovative indicators that are representative of the neonatal intensive care unit target population. This approach moves away from static instruments that are not applicable in the neonatal context and incorporates indicators that are inherent to developmental care.

## 1 Introduction

The stratification of healthcare quality into safe, effective, timely, efficient, equitable, and person-centred underpins efforts to improve it and implies a supportive environment (1).

Quality and patient safety are distinct yet closely related concepts with often overlapping boundaries. Quality in healthcare is a multidimensional concept defined by the WHO as care that is safe, effective, person-centred, timely, efficient, equitable, and integrated. Safety is therefore not external to quality but one of its essential pillars. Patient safety, in contrast, has a more specific operational definition that focuses on the absence of preventable harm and the reduction of unnecessary risk to an acceptable minimum. It is understood as an organised set of activities (processes, behaviours, and technologies) aimed at reducing risk in a consistent and sustainable manner (1).

The environment of the neonatal intensive care unit (NICU) is characterised by demanding specialised care and complex technologies, requiring agile and assertive team practice. The vulnerability of the neonatal patients makes them susceptible to safety incidents with a high potential for harm, necessitating care in complex contexts by differentiated multidisciplinary teams (2–5).

A holistic approach to quality management implies paying attention to all phases of patient care and promoting the reduction of the gap between the intended quality and the quality received and/or perceived by patients. This is achieved through the integration of a planning system and the implementation of quality improvement activities at the different levels of the care system, applying quality and safety concepts in a multifaceted approach (6).

In the context of the NICU, “quality statements” are concise, tangible, and measurable proposals for improving the quality of care for neonatal patients. Methodologies capable of projecting the multidimensional components of neonatal care are essential, as these dimensions influence each other (7).

The need for specific indicators—and their current lack of robustness in the neonatal context—is related to the physiological immaturity of newborns, high technological dependence, and therapeutic complexity, factors that increase vulnerability to safety incidents. Although indicators have been identified for NICUs, they are often based on adult models and fail to capture critical dimensions such as pain, sleep, bonding, and parental involvement (7). The insufficient integration of these relational dimensions limits the scope of the assessment and may render invisible components that are vital for the healthy development of newborns. Despite the use of safety checklists, robust empirical validation of indicators fully aligned with the principles of the DCC remains limited. This shortcoming compromises systematic incorporation into operational tools, such as the Neonatal Patient Safety Checklist (NPSC), and hinders the monitoring of neuroprotective practices. It is safe to say that serious gaps remain in validating content, reliability, and sensitivity to change, particularly regarding experiential and parental indicators (7).

In short, the current challenge is not only to identify indicators but also to ensure that they are derived from structured evidence and are sensitive to the specificities of the NICU so that measurement can be translated into measurable clinical improvement (7).

**Figure 1:**
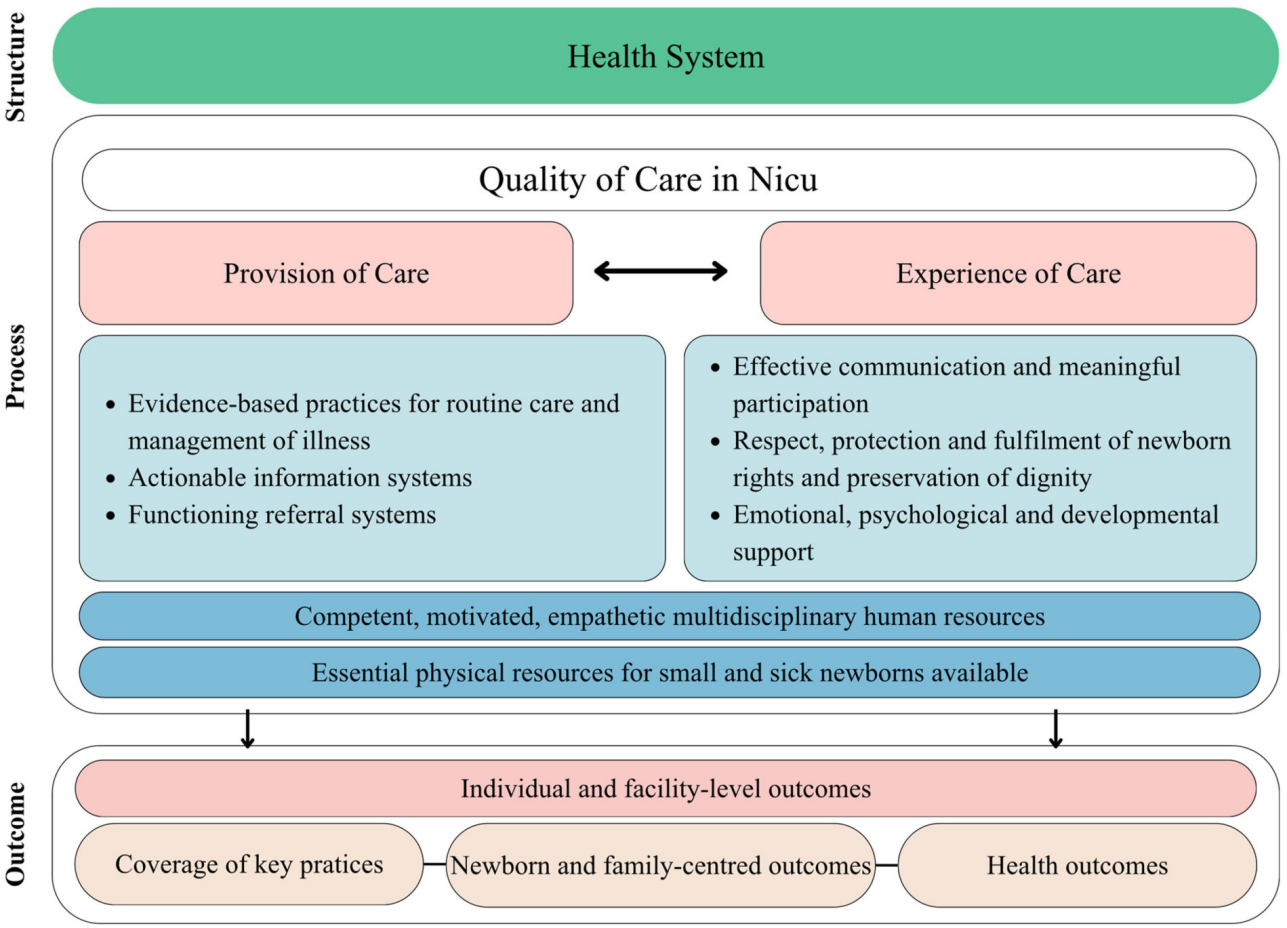
Framework for quality of neonatal care (7).

The framework presented (Figure 1), endorsed by the WHO (7), directly addresses neonatal safety by standardising practices and reducing variability of care in a highly vulnerable population at high risk of harm, mortality, and long-term sequelae (7–9). In line with the WHO quality model, safety is considered a central pillar of quality and is integrated across all levels of care. Organisation into eight domains ensures that care is provided in a consistent, predictable, and evidence-based manner, reducing active and latent failures in neonatal care systems.

Safe care is ensured by evidence-based clinical practices, effective referral systems, and information systems that enable risk identification, event monitoring, and decision support. The experience of care contributes to safety through effective communication and family involvement, which act as additional barriers to error and promote early identification of issues and continuity of care. Health system resources support safety by ensuring proficient, motivated, and empathetic professionals, as well as appropriate neonatal care infrastructure and equipment. The use of the structure–process–outcome model enables the identification of structural vulnerabilities, process failures, and adverse effects on outcomes, reinforcing safety as a measurable outcome of care (7–9).

The integration of family-centred and development-supportive care enhances safety by reducing stress, pain, and exposure to harmful stimuli such as light, noise, and excessive handling, factors recognised as risks for neurodevelopment and adverse events. This framework acts as a risk management and harm prevention tool that promotes safe, consistent, and person-centred neonatal care while contributing to the reduction of adverse events and improving clinical and developmental outcomes (7–9).

To improve neonatal care quality and safety, there is a need for reliable indicators that are valid for their purpose and can capture the full complexity of neonatal care. Studies identifying outcome indicators that are sensitive to care practice have concluded that although indicators have been identified in the context of neonatal intensive care units, many remain generic and are not transferable to a neonatal population (10).

Quality and safety indicators in NICU care are divided into process indicators and outcome indicators (experience of care). While process indicators capture objective information about activities and outcomes related to the provision of care, outcome indicators focus on the patient's experience and are strongly influenced by person-centred care, emphasising indicators related to the neonate, the parent–baby dyad, and overall quality of care (8, 9). However, the subjectivity of outcome (experience of care) indicators means that there are few specific indicators of neonatal patient safety that reflect the uniqueness and specificity of neonatal care.

This is manifested through individualised care with a focus on promoting comfort measures to minimise the impact of negative stimuli from the physical environment of the NICU on neonates and their families. DCC is a dimension of quality and safety that involves understanding the behaviour of the neonatal patient and their parents in their search for safety, planning individualised care, and guiding informed intervention by health professionals (11). In addition to the healthcare professionals’ knowledge and skills, DCC requires coordination between families and care teams, a healthy working environment, and safe supplies (11, 12).

The NPSC operationalises a set of indicators with the strategic aim of assessing sensitive and/or critical points across the continuum of care in the NICU. Its purpose is to strengthen the organisational climate of safety culture and instil a critical awareness of the care provided to neonatal patients under the guarantee of quality and safety. The checklist was designed to reflect the complexity of NICU practice in all actions and interventions. On the one hand, it includes indicators that have been widely validated by the scientific community and implemented in international safety instruments. On the other hand, it is innovative in operationalising safety indicators related to DCC, thereby guaranteeing the quality and safety dimensions advocated by person-centred care (1).

The name NPSC reflects its primary operational focus on preventing errors and harm. However, its inclusion in a quality management framework is justified because it assesses compliance with standards and protocols while implementing measurable indicators to monitor and improve care. Although safety focuses on the prevention of errors and harm, its practical application through tools such as the NPSC is an essential means of achieving overall quality goals, particularly in the neonatal context.

**Figure 2:**
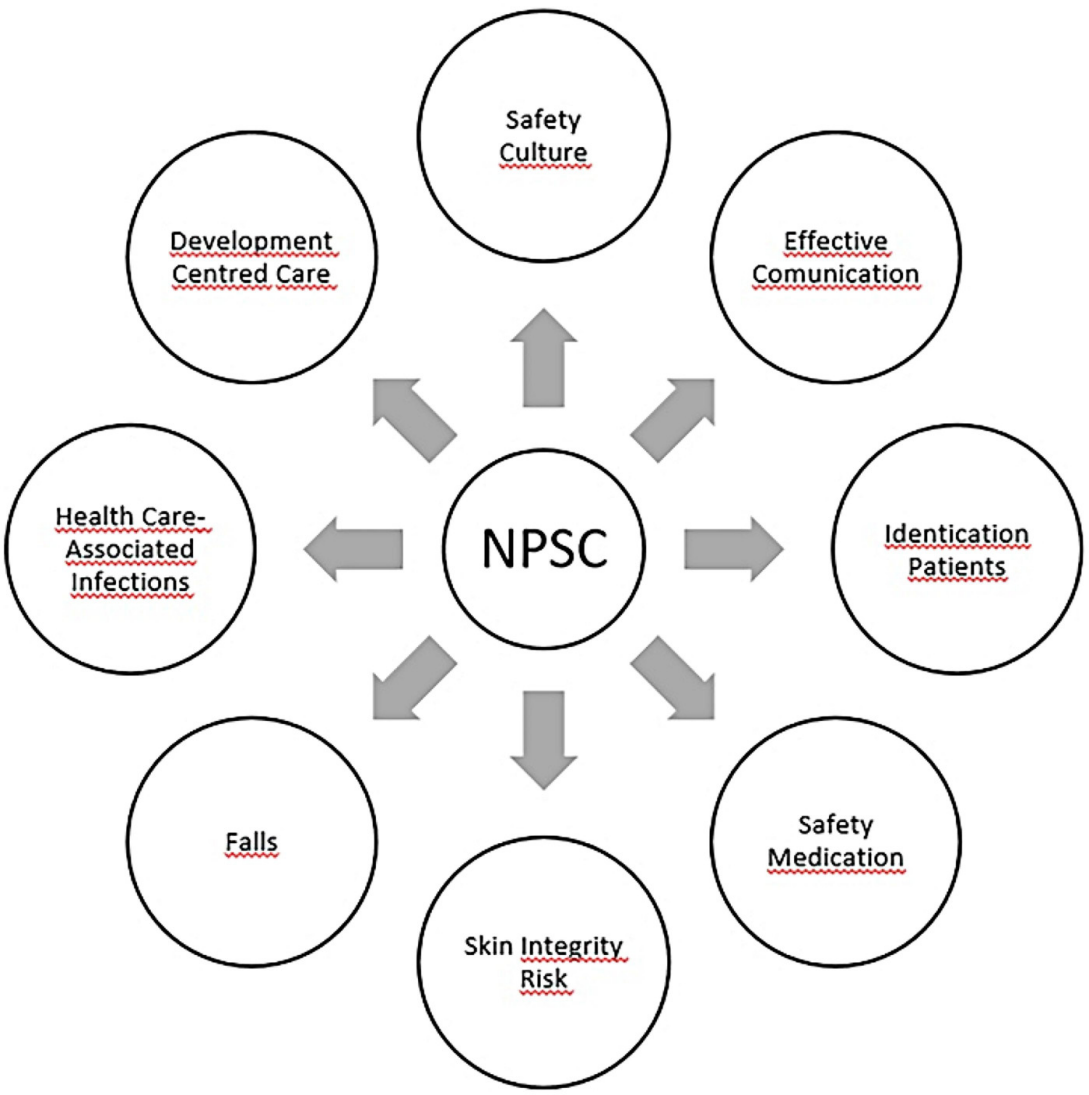
NPSC dimensions care for validation study.

The NPSC is based on well-defined indicators grouped into eight dimensions. These dimensions operationalise a set of indicators adapted to neonatal patients and their families, corresponding to the different moments and areas of action in the continuum of care—from the moment of admission to clinical discharge of the neonatal patient (Figure 2).

The aim of this study was 3-fold: validate the indicators with a panel of national experts, identify and address any gaps, and guarantee a consensus tool that reflects the specific nature of neonatal care.

## 2 Materials and methods

The Delphi method was used for this validation study. This choice reflects the rigour of reaching consensus and determining the validity of new quality indicators related to health systems (13), through a structured communication process among a group of experts that allows collective consensus to be reached (14–17).

A panel of between 15 and 20 experts was selected through a convenience sample of NICU healthcare professionals (doctors and nurses) with expertise in patient safety. To avoid bias and ensure a wide range of expertise, geographically dispersed experts were invited. In addition, an invitation to participate in this study was extended to representatives of neonatal patients in the form of carers represented in a parent support group. After identifying potential experts at the national level, 30 invitations were sent by e-mail, with a 7-day time window to consider responses. Nineteen experts agreed to participate in this study, meeting the inclusion criteria presented in Table 1.

**Table 1:** Expert inclusion criteria.

Category, Subcategory	Description/Distribution
Sample of experts (*n* = 19)	Doctor (*n* = 3)
	Nurse (*n* = 15)
	Parent representative (*n* = 1)
Healthcare experience (healthcare experts *n* = 18)	≥10 years (*n* = 17)
	>5e ≤ (*n* = 1)
Field of work (healthcare experts *n* = 18)	NICU (*n* = 16)
	CQS/Clinical Risk Management (*n* = 11)
	Clinical Supervision (*n* = 18)
	Teaching in Higher Education Institutions (*n* = 3)
Training (health professional experts *n* = 18)	Paediatrics/Neonatology (*n* = 18)
	CDD (NIDCAP and FINE) (*n* = 11)
	Patient safety (*n* = 11)

During this study, the four basic premises of the methodology were respected: guarantee of anonymity, use of a standardised questionnaire adapted for each new round, an interactive process with controlled feedback between the researchers and the experts, and statistical aggregation (14–17).

**Figure 3:**
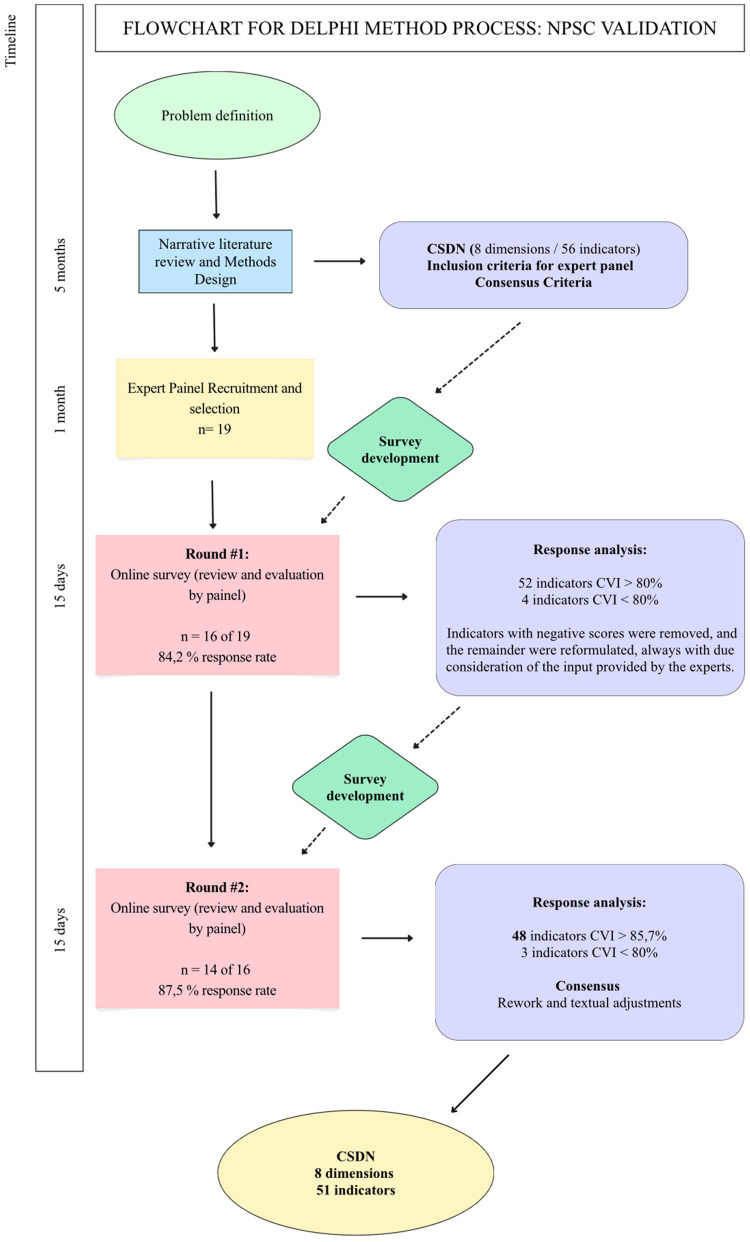
Flowchart for Delphi method process: validation of NPSC [based on Taylor (26)].

This collective construction, as shown in Figure 3, included, as recommended, two rounds of standardised questionnaires with subsequent feedback, encouraging a period of reflection before a new round (14, 18–20). Experts rated the representativeness and relevance of NPSC indicators using a four-point Likert scale (21, 22), with an interquartile range (IQR) of 1 or less being considered a consensus for this study (23). Furthermore, the assessment of indicator representativeness allowed for the calculation of the content validity index (CVI), with values ≥80% deemed acceptable for this study (20, 24–26). The qualitative contributions provided by experts through open-ended responses enabled the integration of textual suggestions, as well as the analysis and conceptual refinement of the indicators.

In Round 1, 19 questionnaires were sent out, and 16 responses were received within the set time frame (15 days after the access link was sent), representing 84.2% of the initial population of experts. Round 2 began with feedback on the data collected in the previous round and a new link to a new electronic questionnaire being sent to the 16 experts, with 14 questionnaires returned (87.5%). The attrition rate between the first and second rounds was 12.5%.

It should be noted that one expert who would have contributed as an end user of the tool (neonatal patient in the form of a carer) contacted the researchers and withdrew from the expert panel, citing difficulties in completing the questionnaire due to its specificity. This represents a limitation in terms of the accessibility of the questionnaire to all stakeholders.

## 3 Results

The first round confirmed that the NPSC contains representative indicators for assessing neonatal patient safety in the NICU and is a robust tool with generally positive results. For example, of the 56 indicators that initially constituted the NPSC, only four obtained a CVI <80%, reflecting reliable operationalisation of the construct (19, 23–25). Indicators that did not reach consensus were removed and the others were reformulated, considering the qualitative input of the experts. Statistical aggregation and interpretation of the data culminated in the reformulation and suppression of indicators, allowing the construction of a new questionnaire with 51 indicators, which was subsequently submitted to the experts in the second round.

The second round aimed to confirm the results obtained and the changes made. The results confirmed the robustness, validity, and representativeness of 48 of the 51 NPSC indicators (94%). By cumulatively assessing consensus and representativeness with an IQR <1 and a variable CVI between 85.71% and 100%, these indicators were considered validated by the expert panel and text adjustments were made (Table 2).

**Table 2:** Main results of the Delphi method applied to the study.

Executive summary			
Neonatal Patient Safety Checklist (NPSC)			
Overall statistics			
Metric	Value	Interpretation	
Total dimensions	8	Complete NPSC structure	
Total indicators	56	Instrument comprehensiveness	
Overall mean CVI	91.6%	Excellent content validity	
Overall mean IQR	0.75	Adequate consensus (≤1)	
Mean agreement (2nd round)	94.6%	Very high consensus	
Reformulated indicators	9	11.5% required adjustments	
Key highlights by dimension			
Category	Dimension	Metric	Observation
Highest CVI	HAIs	100%	Maximum consensus achieved
Lowest IQR	HAIs	0.3	Highest expert consensus
Most complex	DCC	15 indicators	Most extensive dimension
Most reformulated	Communication Safety	3 indicators	Required most adjustments

Methodological note: Validation conducted through Delphi Technique (two rounds) with expert panel. Validation criteria: CVI ≥ 80% and IQR ≤ 1. 94% of indicators validated in second round with high consensus.

Qualitative data were derived from optional text comments and suggestions provided by the 19 experts in the electronic questionnaire associated with each indicator. The qualitative analysis focused on conceptual, semantic, and editorial refinement of the indicators based on direct feedback from the experts. The responses were systematised by the researchers to identify the need for revision, considering only suggestions directly related to the indicator and excluding reports of professional experience to ensure objectivity. These contributions were used to linguistically and structurally reconstruct the instrument, ensuring greater consistency and clarity by reformulating indicators that did not achieve quantitative consensus before moving on to the next round.

It is important to highlight that several dimensions consistently achieved representativeness and consensus across both rounds. Despite the undeniable overall results, it is appropriate to analyse the tool dimensionally and further examine some indicators.

## 4 Discussion

### 4.1 Safety culture

This dimension is composed of indicators with robust measures of central tendency (Me of 4 in 92.3% of the indicators) and an IQR < 1, with representativeness of the indicator fully ensured by CVI values between 81.25% and 100%. Indicators with lower scores reinforce the DGS data (27), highlighting the need for intervention and clarification of issues related to non-punitive responses to errors, reporting, and safe staffing.

### 4.2 Effective communication

In the first round, all indicators with IQR ≤1 and CVI between 81.25% and 93.75% were validated, highlighting the safety of health communication as a key quality factor, promoted by flowcharts, well-defined procedures, good practices, and the involvement of patients and carers. However, the informed consent indicator, despite achieving a CVI of 81.25% in the first round, fell to 78.57% in the second round and was not validated by the panel, although it is recommended by the DGS as a strategy for adapting clinical communication to patient care (27).

In the context of the NICU, family-centred communication allows both the transmission of information and the obtaining of consent and shared decision-making (28), combined with the duty of legal representation of the parents, due to the inability of the neonatal patient to assert the principle of autonomy and provide informed consent for the performance of certain medical, therapeutic, and research procedures (29).

Factors such as the complexity of the NICU setting and the urgency of care, which may justify presumed consent (30), combined with constraints on parental participation in decision-making (31), may explain the lower representativeness attributed to this indicator.

In Portugal, no studies have been identified linking legally required free and informed consent to care in the context of NICUs. Therefore, the conclusions presented here are those of the researchers, stating that informed consent, despite the legal ethical framework that supports it, is absent in NICU care practices, with presumed consent prevailing. In view of this, and contrary to what was defined regarding the exclusion of indicators that did not cumulatively obtain scores within the established criteria, the researchers decided to retain the following indicator in the NPSC: “Informed, free and informed consent is guaranteed through registration and justification in the neonatal patient's clinical record.”

### 4.3 Neonatal patient identification

Neonatal patients are known to be vulnerable to safety incidents due to their uniqueness, requiring organisations to implement and operationalise well-defined standardised norms and strategies (27, 32, 33). The investment and organisational consolidation in team practices regarding awareness and implementation of strategies for unambiguous patient identification (28) was confirmed by the results of the study when validating six out of seven indicators (85.7%) that make up the NPSC dimension of unambiguous identification of neonatal patients, with CVI ≥ 87.5% and IQR < 1.

### 4.4 Safety medication

All eight indicators presented to the expert panel in the first round had an IQR < 1, guaranteeing their consensus. However, regarding the CVI, the indicator related to therapeutic reconciliation (27, 34) was considered unrepresentative, with a value of 75%. This asymmetry of results (consensual but not very representative) led to its reformulation and reassessment in the second round, after having been validated with an IQR = 0 and a CVI of 85.7%.

This outcome confirms one of the advantages of the Delphi method: By forcing experts to complete several rounds of questionnaires, it encourages and improves reflected consensus and the construction of a coherent and robust instrument (14, 18–20).

### 4.5 Skin integrity risk

Regarding the skin integrity dimension, all indicators achieved consensus, as evidenced by measures of central tendency and dispersion (IQR < 1) and by CVI values ranging from 81.25% to 93.75%.

It is important to emphasise the need to use skin integrity risk assessment scales that have been validated and parameterised for neonatal patients (35–37), as the instrument adopted by the General Health Directorate (DGS) suggests a reductive nature and needs to be updated, as it includes children older than 28 days (38).

### 4.6 Neonatal fall risk

All three indicators related to the risk of neonatal falls obtained an IQR = 0 and a CVI > 87.5% in the first round. The analysis of the qualitative data dictated the reformulation of the indicators and their submission to the second round. The indicator related to the use of a scale to assess the risk of neonatal falls was eliminated by the experts. Nevertheless, this risk is included in the NPSP (27), even though there is still no parameterised scale for neonatal patients validated for the Portuguese population, which may have led to the difference in results between rounds. In this context, and given the lack of validated scales for the Portuguese neonatal population, the indicator was reformulated to include only the assessment of fall risk, without reference to a specific scale. While this finding may reflect a limitation and/or fragility of the final instrument, it is considered dynamic and evolving, and a scale can be associated later.

### 4.7 Healthcare-associated infections

This was the most robust and consistent dimension in the NPSC, achieving the highest levels of consensus and representativeness from the expert panel. This reflects the impact of economic investment and international and national initiatives aimed at increasing the literacy and pedagogical capacity of care providers (1, 27, 39).

### 4.8 Development-Centred care

DCC indicators represents an innovative component of the instrument, making the NPSC unique in its approach to assessing the quality and safety of neonatal patients in NICUs. This innovation introduced greater complexity in validating the consensus and representativeness of its 15 indicators, which encompass a range of elements related to safe and parameterised environments for neonatal patients. These focus on improving the cognitive and behavioural development of neonates, including redesigning the NICU environment, individualisation of care, family-centred care, non-nutritive sucking, appropriate positioning and restraint, early initiation of breastfeeding, pain control, and kangaroo care (40). Only two indicators failed to achieve consensus and representativeness, representing an important milestone in terms of individualised neonatal care and underscoring the robustness and consolidation of the NPSC as a parameterised tool for use in NICUs.

The first failed indicator consists of the physical design of the NICU, with an IQR > 1 and a CVI of 75%. It is more difficult to change and incorporate into NICU practice, although it is supported by the scientific community (41); it was therefore reformulated and presented in the second round, presenting data that cumulatively reached consensus among experts.

The second failed indicator relates to the time of discharge, linked to the provision of a scale of self-perceived parenting skills to ensure safety through parenting training (42–47). This indicator failed to achieve consensus among experts in either round regarding its validity (CVI < 70%) and/or representativeness (IQR = 2).

The involvement and training of caregivers in the pre-discharge phase is essential to improve patient safety (9, 11, 41, 48), with the Discharge Guide being a tool to operationalise and consolidate parental knowledge and skills acquired during hospitalisation in the NICU. The scale aims to validate this knowledge and contribute to the safest possible transition to home care (42–47). Because of its importance, and despite the previously defined criteria, this indicator was not removed, but was reformulated considering expert suggestions, with the statement referring to the timely planning of discharge, allowing caregivers to ask questions and validate the teaching. The non-use of a reference scale seems to be a limitation of the NPSC and would benefit from further studies.

## 5 Strengths and limitations of the study

Given its innovative nature, the consensus reached on indicators related to DCC is particularly relevant, as these were previously absent from similar instruments, which prevented their assessment. DCC, as a holistic approach that respects the individual rhythms and needs of newborns and their families, has a significant impact on health outcomes. In this context, the integration of these indicators is a relevant contribution to the evaluation and monitoring of this approach to care.

However, it is important to reflect on the reluctance of specialists to validate the indicator of free and informed consent. This resistance seems to be associated with the difficulty of care providers in distancing themselves from the biomedical model, as well as their reluctance to involve the patient/caregiver in decision-making. This finding highlights the need to deepen our understanding of this phenomenon.

It is therefore necessary to conduct further studies that enable us to understand these dynamics and to outline strategic axes focused on neonatal patients and their families. A potential limitation of this study may lie in the failure to consider the perspectives of parents and carers, whose involvement is crucial. As end users of healthcare, patients and their families have a unique perspective on outcomes and should be included in patient safety planning to ensure a truly patient-centred approach.

## 6 Conclusions

This study enabled the validation of a set of relevant indicators applicable to NICU practice, addressing the challenge inherent to the validation of indicators sensitive to neonatal care, which are often marked by their subjective nature (9).

The rigour of the methodological process, combined with the structured integration of the best scientific evidence in the construction of the indicators, culminated in the development of a tool with high credibility and reliability. Its innovative nature lies precisely in its ability to overcome the “static” and reductive approaches traditionally associated with quality assessment, moving closer to DCC-oriented indicators and reflecting the complexity, interdependence, and dynamism inherent in neonatal practice.

Thus, at the end of this study, NPSC asserts itself not only as a valid instrument but also as a methodological innovation in the field of continuous quality improvement, with potential for extrapolation to different NICUs and an effective capacity to promote sustained gains in the safety and quality of neonatal care.

## Data Availability

The raw data supporting the conclusions of this article will be made available by the authors without undue reservation.

## References

[1] WHO. Handbook for National Quality Policy and Strategy: A Practical Approach for Developing Policy and Strategy to Improve Quality of Care. Geneva: World Health Organization (2020).

[2] Chatziioannidis I Mitsiakos G Vouzas F. Focusing on patient safety in the neonatal intensive care unit environment. J Pediatr Neonatal Individ Med (JPNIM). (2017) 6(1):e060132. 10.7363/060132

[3] Lage MJ. Segurança do doente: da teoria à prática clínica. Revista Portuguesa de Saúde Pública. (2010) (10):11–6.

[4] Fragata J Martins L. *O erro em medicina*. Coimbra: Almedina (2014).

[5] Silva EMB Garcia CRF Silva DM Duarte JC. A segurança dos cuidados da criança hospitalizada: perceção dos enfermeiros. Revista de Psicol Criança e Adolesc J Child Adolescent Psychol. (2018) 9:1. 10.34628/sary-e786

[6] Sampath B Rakover J Baldoza K Mate K Lenoci-Edwards J Barker P. Whole System Quality: A Unified Approach to Building Responsive, Resilient Health Care Sys-tems. IHIWhite Paper. Boston: Institute for Healthcare Improvement. (Disponível em www.ihi.org) (2021).

[7] WHO. Standards for Improving Quality of Care for Small and Sick Newborns in Health Facilities. Geneva: World Health Organization (2020b).

[8] Wang D Sacks E Odiase OJ Kapula N Sarakki A Munson E A scoping review, mapping, and prioritisation process for emergency obstetric and neonatal quality of care indicators: focus on provision and experience of care. J Glob Health. (2023) 13:04092. 10.7189/jogh.13.04092 37824168 PMC10569369

[9] Benova L Moller AB Moran AC. What gets measured better gets done better": the landscape of validation of global maternal and newborn health indicators through key informant interviews. PLoS One. (2019) 14(11):e0224746. 10.1371/journal.pone.0224746 31689328 PMC6830807

[10] Saturno-Hernández PJ Martínez-Nicolás I Moreno-Zegbe E Fernández-Elorriaga M Poblano-Verástegui O. Indicators for monitoring maternal and neonatal quality care: a systematic review. BMC Pregnancy Childbirth. (2019) 19(1):25. 10.1186/s12884-019-2173-2 30634946 PMC6330388

[11] Babaie M Nourian M Atashzadeh-Shoorideh F Manoochehri H Nasiri M. Patient safety culture in neonatal intensive care units: a qualitative content analysis. Front Public Health. (2023) 11:1065522. 10.3389/fpubh.2023.1065522 36741949 PMC9895384

[12] Hagen IH Svindseth MF Nesset E Orner R Iversen VC. Validation of the neonatal satisfaction survey (NSS-8) in six Norwegian neonatal intensive care units: a quantitative cross-sectional study. BMC Health Serv Res. (2018) 18(1):222. 10.1186/s12913-018-3031-z 29587812 PMC5872573

[13] Zarili TFT Castanheira ERL Nunes LO Sanine PR Carrapato JFL Machado DF Técnica Delphi no processo de validação do Questionário de Avaliação da Atenção Básica (QualiAB) para aplicação nacional. Saúde e Sociedade. (2021) 30:e190505. 10.1590/S0104-12902021190505

[14] Marques JBV de Freitas D. The Delphi method: characterization and potentialities for educational research. Pro-Posições. (2018) 29(2):389. 10.1590/1980-6248-2015-0140

[15] Humphrey-Murto S Wood T Gonsalves C Mascioli K Varpio L. The Delphi method. Acad Med. (2020) 95(1):168. 10.1097/ACM.0000000000002887 31335812

[16] Niederberger M Spranger J. Delphi technique in health sciences: a map. Front Public Health. (2020) 8:457. 10.3389/fpubh.2020.00457 33072683 PMC7536299

[17] Barrios M Guilera G Nuño L Gómez-Benito J. Consensus in the Delphi method: what makes a decision change? Technol Forecast Soc Change. (2021) 163:120484. 10.1016/j.techfore.2020.120484

[18] Barrett D Heale R. What are Delphi studies? Evidence Based Nursing. (2020) 23(3):68–9. 10.1136/ebnurs-2020-103303 32430290

[19] Lacasta MJ Cardona A Vidal-Lancis C Carles-Lavila M Group P. Development of support material for health professionals who are implementing shared decision-making in breast cancer screening: validation using the Delphi technique. BMJ Open. (2022) 12(2):e052566. 10.1136/bmjopen-2021-052566 35105575 PMC8808455

[20] Spranger J Homberg A Sonnberger M Niederberger M. Reporting guidelines for Delphi techniques in health sciences: a methodological review. Zeitschrift für Evidenz, Fortbildung und Qualität im Gesundheitswesen. (2022) 172:1–11. 10.1016/j.zefq.2022.04.025 35718726

[21] Alexandre NMC Coluci MZO. Validade de conteúdo nos processos de construção e adaptação de instrumentos de medidas. Ciência & Saúde Coletiva. (2011) 16:3061–8. 10.1590/S1413-81232011000800006 21808894

[22] Campbell M Katikireddi SV Sowden A McKenzie JE Thomson H. Improving conduct and reporting of narrative synthesis of quantitative data (ICONS-Quant): protocol for a mixed methods study to develop a reporting guideline. BMJ open. (2018) 8:2. doi: 10.1136%2Fbmjopen-2017-020064

[23] Pedreira RB Rocha SV Santos CA Vasconcelos LR Reis MC. Content validity of the geriatric health assessment instrument. Einstein (São Paulo). (2016) 14(2):158–77. 10.1590/S1679-45082016AO3455 27462889 PMC4943349

[24] Shang Z. Use of Delphi in health sciences research: a narrative review. Medicine (Baltimore). (2023) 102(7):e32829. 10.1097/MD.0000000000032829 36800594 PMC9936053

[25] Martins PR Fonseca LF Rossetto EG. Developing and validating the perioperative thirst discomfort scale. Revista da Escola de Enfermagem da USP. (2017) 51:e03240. 10.1590/S1980-220X2016029003240 28746560

[26] Taylor E. We agree, don't we? The Delphi method for health environments research. HERD: Health Environments Research & Design Journal. (2020) 13(1):11–23. 10.1177/1937586719887709 31887097

[27] DGS. Documento Técnico Para a Implementação do Plano Nacional Para a Segurança dos Doentes. Lisboa: Direção Geral de Saúde (2022).

[28] Labrie NHM van Veenendaal NR Ludolph RA Ket JCF van der Schoor SRD van Kempen AAMW. Effects of parent-provider communication during infant hospitalization in the NICU on parents: a systematic review with meta-synthesis and narrative synthesis. Patient Educ Couns. (2021) 104(7):1526–52. 10.1016/j.pec.2021.04.023 33994019

[29] OE. Guia Orientador de Boa Prática—Adaptação à parentalidade durante a hospitalização. Mesa do Colégio da Especialidade de Enfermagem de Saúde Infantil e Pediátrica (MCEESIP). Ordem dos Enfermeiros. ISBN: 978-989-8444-26-4 (2015).

[30] Direção-Geral da Saúde (DGS). Norma nº015/2013: Consentimento Informado, Esclarecido e Livre Dado por Escrito. Lisboa: Direção-Geral da Saúde (2015). Available online at: https://www.dgs.pt/normas-orientacoes-e-informacoes/normas-e-circulares-normativas/norma-n-0152013-de-03102013.aspx

[31] Parish O Williams D Odd D Joseph-Williams N. Barriers and facilitators to shared decision-making in neonatal medicine: a systematic review and thematic synthesis of parental perceptions. Patient Educ Couns. (2022) 105(5):1101–14. 10.1016/j.pec.2021.08.033 34503868

[32] de Souza Gomes AP Querido DL da Silva GR de Almeida LF Rocha RG. Identificação do paciente em neonatologia para assistência segura. Cogitare Enfermagem. (2017) 22(3):e49501. 10.5380/ce.v22i3.49501

[33] JCI. Metas Nacionais de Segurança do Paciente (NPSGs). Joint Comission Internacional. Available online at: https://www.jointcommission.org/standards/national-patient-safety-goals/hospital-national-patient-safety-goals/ (2023).

[34] DGS. Norma 018/2016: Reconciliação da Medicação. Lisboa: Direção Geral da Saúde (2016).

[35] Ferreira EMC Pereira ARDC Montoito AIM Curado MADS. Clinical validation of the neonatal skin condition score with Portuguese newborns. Revista Gaúcha de Enfermagem. (2023) 44:e20220059. 10.1590/1983-1447.2023.20220059.en 37377269

[36] Martins COA Curado MA. Observation neonatal skin risk assessment scale: statistical validation with newborns. Revista de Enfermagem Referência. (2017) 4(13):43. 10.12707/RIV16082

[37] Silva RSLPD. Avaliação do risco de lesão da pele do recém-nascido internado em unidade de cuidados intensivos neonatais: validação da escala ISSA para a população portuguesa (Doctoral dissertation). Available online at: http://hdl.handle.net/10400.19/8366 (2024).

[38] DGS. Norma 017/2011: Escala de Braden: Versão Adulto e Pediátrica (Braden Q). Direção Geral de Saúde. Available online at: https://www.dgs.pt/departamento-da-qualidade-na-saude/ficheiros-anexos/orientacao_ulceraspdf-pdf.aspx (2011b).

[39] Slawomirski L Klazinga N. The economics of patient safety: from analysis to action. Organisation for Economic Co-operation and Development; Available online at: https://one.oecd.org/document/DELSA/HEA/WD/HWP(2022)13/en/pdf (2022).

[40] Raghu VA Vatsa M. Early neurodevelopmental supportive care: approach to enhance the neurodevelopmental outcome in premature and low birth weight infant. J Integr Health Sci. (2021) 9(2):99–106. 10.4103/jihs.jihs_24_21

[41] EFCNI. Standards Europeus de Cuidados de Saúde ao Recém-nascido: Folheto informativo. Munich, Germany. Disponível em Available online at: https://newborn-health-standards.org/wp-content/uploads/2022/05/2022_05_18_ESCNH_Information-brochure_PT.pdf (2022).

[42] Marques S Sá M. Competências maternas auto-percebidas no contexto da prematuridade. Revista Referência. (2004) 11:33–41. Available online at: https://rr.esenfc.pt/rr/index.php?module=rr&target=publicationDetails&pesquisa=&id_artigo=36&id_revista=5&id_edicao=10

[43] Rodrigues S. Supervisão em Enfermagem Neonatal: pais e enfermeiros como parceiros no desenvolvimento de competências; Dissertação de Mestrado em Supervisão; Aveiro: Universidade de Aveiro—Departamento de Didática e Tecnologia Educativa. Acedido em Available online at: http://ria.ua.pt/handle/10773/1416 (2010).

[44] Wittkowski A Garrett C Calam R Weisberg D. Self-report measures of parental self-efficacy: a systematic review of the current literature. J Child Fam Stud. (2017) 26(11):2960–78. 10.1007/s10826-017-0830-5 29081640 PMC5646137

[45] Monteiro F Fonseca A Pereira M Canavarro MC. Perceived maternal parenting self-efficacy scale: factor structure and psychometric properties among Portuguese postpartum women. Midwifery. (2022) 105:103240. 10.1016/j.midw.2021.103240 34971870

[46] Schneider J Harari MM Faure N Lacroix A Borghini A Tolsa JF Joint observation in NICU (JOIN): a randomized controlled trial testing an early, one-session intervention during preterm care to improve perceived maternal self-efficacy and other mental health outcomes. PLoS One. (2024) 19(4):e0301594. 10.1371/journal.pone.0301594 38662661 PMC11045081

[47] EFCNI. Infant- & family-centered development care. European standards care for newborn health. European Foundation for the Care of Newborn Infants Available online at: https://newborn-health-standards.org/wp-content/uploads/2021/09/27-TEG_IFCDC_complete.pdf (2021).

[48] WHO. Global Patient Safety Action Plan 2021–2030: Towards Eliminating Avoidable Harm in Health Care. Geneva: World Health Organization (2021).

